# Radiotranscriptomics identified new mRNAs and miRNA markers for distinguishing prostate cancer from benign prostatic hyperplasia

**DOI:** 10.1002/cam4.6728

**Published:** 2023-11-21

**Authors:** Qian Yang, Qiuyang Li, Nan Li, Dingyi Wang, Shaoxi Niu, Peng Tang, Jing Xiao, Jiahang Zhao, Pei Wang, Yukun Luo, Jie Tang

**Affiliations:** ^1^ Department of Ultrasound, Air Force Medical Center PLA, Air Force Military Medical University Beijing China; ^2^ Department of Ultrasound First Medical Center, Chinese PLA General Hospital Beijing China; ^3^ Department of Urology, First Medical Center Chinese PLA General Hospital Beijing China; ^4^ Department of Orthopedics, China Rehabilitation Research Center Beijing Charity Hospital Beijing China; ^5^ Department of Ultrasound Diagnosis and Treatment Center Xi'an International Medical Center Hospital Xian China

**Keywords:** prostate cancer, radiomics, radiotranscriptomics, ultrasound

## Abstract

The present study investigated ultrasound (US) phenotypes reflecting prostate cancer (PCa)‐related genetic mutations. Herein, integration of radiotranscriptomic data, US and contrast‐enhanced ultrasound (CEUS) radiomic images, and RNA sequencing was performed with the aim of significantly improving the accuracy of PCa prognosis. We performed radiotranscriptomic analysis of clinical, imaging, and two genomic (mRNA and microRNA expression) datasets from 48 and 22 men with PCa and benign prostatic hyperplasia (BPH), respectively. Twenty‐three US texture features and four microvascular perfusion features were associated with various patterns of 52 differentially expressed genes related to PCa (*p* < 0.05); 17 overexpressed genes were associated with two key texture features. Twelve overexpressed genes were identified using microvascular perfusion features. Furthermore, mRNA and miRNA biomarkers could be used to distinguish between PCa and BPH. Compared with RNA sequencing, B‐mode and CEUS features reflected genomic alterations associated with hormone receptor status, angiogenesis, and prognosis in patients with PCa. These findings indicate the potential of US to assess biomarker levels in patients with PCa.

## Abstract 2

1. Our radiotranscriptomics investigations of PCa provided three ultrasound key features identified with radiomics: two texture features and one microvascular perfusion feature. 2. The following co‐regulated, differentially expressed mRNAs and microRNAs were found in three datasets related to prostate cancer: SNAI2, CAV1, RD5A2, RND3, and miR205‐5p, miR205‐3p, miR221‐3p. 3. The enriched functions in the three key features of the significant genes included negative regulation of epithelial cell proliferation, the androgen metabolic process, male gonad development, and the lipid biosynthetic process.

## 1 INTRODUCTION

The worldwide prostate cancer (PCa) burden is expected to grow to approximately 2.3 million new cases and 740,000 deaths by 2040 due to the aging of the population.^1^ The incidence of PCa is rapidly increasing in response to economic development, prolonged life expectancy, and adaptation to Western lifestyles in Asia.^2^ The complexities of primary PCa diagnosis are rooted in the multifocal nature of the disease. Numerous reports have documented that >80% of primary PCa cases show multiple topographically and histomorphologically distinct tumor foci.^3^ Next‐generation sequencing has shown high levels of genomic diversity among patients (interpatient heterogeneity), within a given primary tumor (intratumoral heterogeneity), and between distinct tumor lesions and different metastatic sites (intertumoral heterogeneity).^4^

Radiomics can be used to mine high‐throughput quantitative and noninvasive imaging features, such as heterogeneity or asymmetrical enhancement, to improve cancer diagnosis and treatment.^5^ Radiotranscriptomics^6^ is a branch of radiogenomics that combines “radiomics” and “transcriptomics”, including the expression level of messenger RNA (mRNA), microRNA (miRNA), and long noncoding RNA, which can also be useful in the search for potential tumor biomarkers.^7^ The rationale behind the implementation of a new multiparametric approach (i.e., combining the information from complementary biomarkers such as tissue texture, elasticity, or perfusion, with both magnetic resonance imaging and ultrasound) is to overcome the complexity of PCa as a multifocal and heterogeneous disease. Several advances in US techniques have improved the detection of PCa. Identifying imaging features associated with a high risk of clinically significant disease that are functionally related to defined molecular alterations will ultimately allow the detection of molecular heterogeneity within tumors and provide an exciting new intersection between imaging and transcriptomics. Combined with molecular imaging, these methods will significantly improve tissue sampling and increase the accuracy of precision medicine for treating localized PCa.

We assumed that the morphology and vascular phenotypes visualized with US could be correlated with the expression of specific genes reflecting the growth or angiogenesis in PCa and that radiotranscriptomic signatures at the imaging level could capture the underlying intratumor heterogeneity at the molecular level. Several radiotranscriptomic^8^, ^9^, ^10^ investigations have described the association between PCa features and genetic alterations using magnetic resonance imaging but rarely US.^11^ This study underscores the potential of combining traditional clinical features with radiotranscriptomics. By identifying specific biomarkers, our study suggests that the accuracy of predicting prostate cancer (PCa) can be enhanced. Our findings focused on the radiotranscriptomics‐based identification of new biomarkers that can distinguish prostate cancer from benign prostatic hyperplasia.

## 2 MATERIALS AND METHODS

### 2.1 Workflow of transcriptomics and radiomic data approach

**FIGURE 1:**
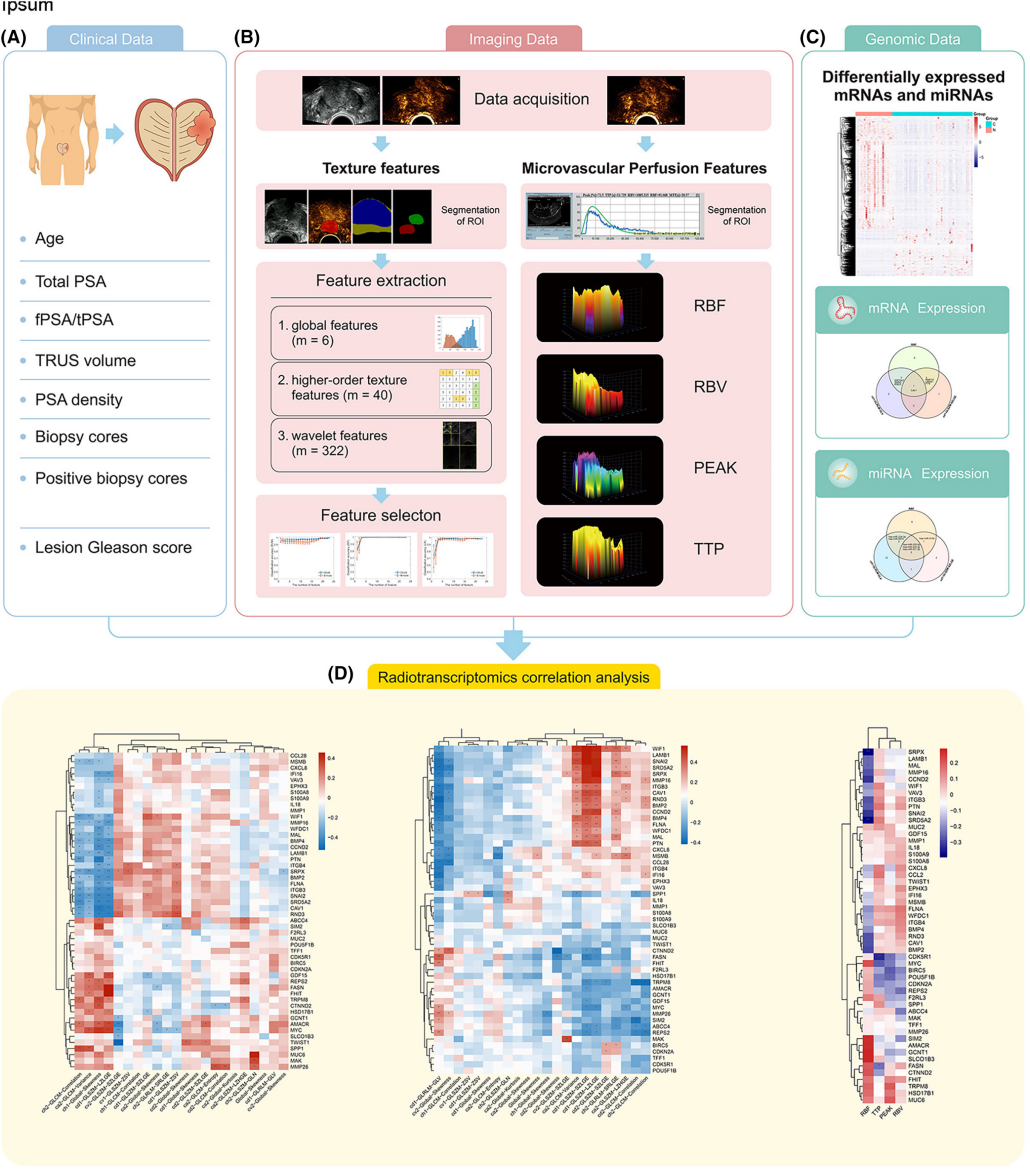
Experimental design. (A) Clinical data (blue panel); (B) Imaging data (radiomic analysis) (pink panel); (C) Differentially expressed mRNA and miRNAs (green panel); (D) The final step is to link radiomic information to transcriptome data (yellow panel).

The radiotranscriptomic workflow is shown in Figure 1. It illustrates our integrated approach to jointly analyzing clinical, imaging, and transcriptomic data from patients with PCa. Radiomic analysis included texture and microvascular perfusion features, which were used to extract imaging features from the PCa tumor region. Following the guidelines and reporting recommendations of IBSI helps ensure that the texture features are reproducible and comparable.^12^, ^13^ The types, parameter settings, and value range of gray intensity and texture features in this study were implemented using a Medical Interaction Toolkit (MITK) (https://docs.mitk.org/2021.02/) for the manual delineation of tumor boundaries and identification of tumor regions of interest (ROI) for radiomics analysis.^14^ This ensured the reproducibility and comparability of the results. The specific methods are presented in Supplemental Materials. The microvascular perfusion features of CEUS images and video clips were analyzed using the QontraX software 4.00 (Esaote), and whole‐image parameter analysis was performed on a manually drawn reference plane (*pink panel*) for perfusion within a specific ROI in the entire region of the selected in‐frame tumor. Tissues from the identified targets were obtained for the analysis of differentially expressed genes (DEGs) using next‐generation sequencing (*green panel*) (Gene Denovo Biotechnology Co., Ltd). The final step was linking the radiomic information to the transcriptome data to identify high‐performance candidate imaging features (*yellow panel*).

### 2.2 Study participant characteristics

From September 2020 to July 2021, 70 consecutive participants with suspected PCa were scheduled for US‐guided biopsy; 22 tissue samples were benign and 48 were malignant. These were used to evaluate the B‐mode and CEUS values to distinguish benign from malignant masses. B‐mode and CEUS values were used to evaluate the benign and malignant masses. The inclusion criteria included: (i) patients with clinical symptoms (frequent urination, urgency, or dysuria pain) or prostate‐specific antigen (PSA) level >4 ng/mL; (ii) transrectal B‐mode US and CEUS examinations were performed before US‐guided biopsy; (iii) pathological results were confirmed by biopsy. The exclusion criteria included: (i) allergy to US contrast agents (*n* = 1); (ii) inadequate image quality of US and CEUS scans (*n* = 21); or (iii) inadequate gene sequencing samples (*n* = 3).

### 2.3 US imaging acquisition

Voxel size is one of the most important imaging parameters that varies significantly depending on the modality, vendor, and imaging protocols used. To reduce the influence of US resolution on voxels, we uniformly fixed the imaging parameters of ultrasound images. We used a MyLab Twice US system (Esaote SpA) with an EC1123 transrectal probe (frequency: 3–9 MHz; contrast frequency: 3–6 MHz), mechanical index of 0.04–0.13, gain adjustment: 66%, depth: 53 mm, and using X‐View adaptive imaging technology (automatic removal of intelligent speckle noise in the whole process, including intelligent sound beam adjustment, signal speckle noise suppression, pixel optimization adjustment, and other technologies for improved imaging quality). Abnormal echo patterns (calcifications, cysts, and hypoechoic lesions) of the prostate were recorded. B‐mode and CEUS examinations were performed by two radiologists who analyzed the imaging phenotypes. A senior sonographer (>10 years of experience) performed manual delineation of the lesion boundary and determined that the ROI minimized the influence of subjective differences. Another senior sonographer (>10 years of experience) confirmed the delineation of the lesion area. In cases of disagreement, the two sonographers discussed the issue and reached an agreement. If a patient had multiple suspicious lesions, the largest lesion was used as an ROI for further evaluation. The US contrast agent SonoVue (Bracco SpaA) consisted of microbubbles filled with 2.5 μm sulfur hexafluoride. For CEUS examination, a rapid intravenous bolus (4.8 mL) of SonoVue was administered, followed by irrigation with 5 mL of normal saline. Continuous real‐time digital imaging was performed immediately after injection for 5 min. The imaging data were retrospectively analyzed.

### 2.4 Prostate biopsy procedure and pathology examination

The US instrument scanner and transrectal probe used in prostate biopsy were consistent with TRUS. Biopsy was performed using an 18‐G biopsy gun (Bard Biopsy Systems, Tempe, AZ, USA) with a length of 18 mm and a penetration depth of 22 mm. In this study, we used multiple MRI scans to determine whether the tumor protruded from the exocapsular growth or invaded the seminal vesicle prior to the biopsy procedure. Transrectal ultrasound (TRUS) examinations were performed by employing blanket transperineal biopsies to reduce infection‐related complications. The lesion selected as the ROI was then biopsied. All patients underwent the “12 + X” biopsy, a targeted biopsy for suspicious areas (combined with B‐mode and CEUS) based on a 12‐core transrectal systematic biopsy performed by one radiologist with more than 10 years of biopsy experience. The needles were injected into the cognitive suspicious targeted nodules. The cognitive targeted biopsy was performed as follows: first, the radiologist reviewed the MRI results; and second, the radiologist used the US information to perform the targeted biopsy for the most remarkable suspicious nodules guided by US images. In our study, tissue biopsy specimens were collected with two biopsy cores in each region. One specimen was used for pathological diagnosis, while the other was flash frozen in liquid nitrogen for next‐generation sequencing (mRNA and miRNAs). We recorded spatial locations of each biopsy site in detail and used the size and pathological findings to determine the location and nature of the lesion, thus ensuring that the description of pathological locations matched the relevant lesions on TRUS images. Regarding pathological results, all histopathologic specimens were labeled as cancer or benign prostatic hyperplasia lesions using a combination of primary and secondary Gleason score by a pathologist. For each biopsy core, the maximum Gleason score was recorded.

### 2.5 mRNA and miRNA sequencing and analysis

Total RNA was prepared using the Trizol reagent (Invitrogen). The quality of RNA samples was assessed with an Agilent 2100 Bioanalyzer (Agilent Technologies). Total RNA (1 μg per sample) was used for the construction of a sequencing library. Eukaryotic mRNAs were enriched by Oligo(dT) beads and by removing prokaryotic mRNAs and rRNAs using the Ribo‐ZeroTM Magnetic Kit (Epicenter). Enriched mRNAs were then fragmented using a fragmentation buffer and reverse transcribed into cDNAs with random primers. After synthesis, cDNAs were purified using the QiaQuick PCR extraction kit (Qiagen), ligated to Illumina sequencing adapters, selected by gel electrophoresis, and amplified by PCR. Subsequently, cDNA libraries were sequenced using the Illumina Novaseq 6000 by Guangzhou GENE DENOVO Biology (Co. Ltd.). For miRNA sequencing, RNA fragments of 18–30 nucleotides in length were enriched by polyacrylamide gel electrophoresis (PAGE). After adding 3′ and 5′ adapters, samples were subjected to RT‐PCR, and PCR products with 140–160 bp size were enriched to generate a cDNA library and sequenced using the Illumina Novaseq 6000.

DEGs were assessed by analyzing differential RNA expression between the two groups. Transcripts with a *p*‐value <0.05 and an absolute fold change of ≥2 were considered differentially expressed. Namely, miRNAs that exhibited an absolute fold change of ≥1.5 and a *p*‐value <0.05 were classified as differentially expressed. Correlations between US imaging phenotypes and gene expression were analyzed using Pearson's correlation coefficients and the R package psych (Version 2.2.3) (http://personality‐project.org/r/psych/HowTo/getting_started.pdf). The correlations between the miRNAs and their co‐expressed mRNA were analyzed using a Sankey diagram. Cytoscape software (version 3.7.1; http://www. cytoscape.org/) and the ggalluvial R package (https://cran.r‐project.org/web/packages/ggalluvial/vignettes/ggalluvial.html) were used to visualize the Sankey diagram. Gene Ontology (GO) (http://geneontology.org/) and Kyoto Encyclopedia of Enrichment Genes and Genomes (KEGG) (https://www.genome.jp/kegg/) pathway analyses were performed using the GO and KEGG databases. The calculated *p*‐value was corrected for false discovery rate, using a false discovery rate of 0.05 as a threshold. The real‐time interactive online data analysis platform OmicSmart (http://www.omicsmart.com) was used for bioinformatics analysis. The RNA‐seq data generated in this research have been placed in the NCBI Sequence Read Archive database under the accession code PRJNA916752 (http://www.ncbi.nlm.nih.gov/bioproject/PRJNA916752).

### 2.6 Statistical analysis

In the model validation phase, we tested and compared the performance of three machine learning predictive models (random forest, naïve Bayes, and SVM) with four datasets, including clinical features, molecular biomarkers, radiomics features, and the combined datasets. Data were analyzed using GraphPad Prism, v9.0.2 (GraphPad Software), and summarized as the mean ± SD. Correlation analysis was performed using Pearson analysis. Unpaired two‐tailed Student's *t*‐tests or analysis of variance were used to compare two or more groups, as indicated in the figure legends.

## 3 RESULTS

### 3.1 Clinical and transcriptomic data collected from patients with Pca and BPH enabled radiotranscriptomic analysis

We processed the clinical data (age, total PSA, ratio of free to total PSA, prostate volume, PSA density, prostate biopsy pathology, biopsy cores, positive biopsy cores, Gleason score, and pathological stage) and two types of transcriptomic (mRNA and miRNA) data collected from 48 patients with primary PCa tumors and 22 patients with benign prostatic hyperplasia (BPH). PSA density was calculated as total PSA/prostatic volume. Table 1 summarizes the clinical and pathological characteristics of participants.

**TABLE 1:** Characteristics of the patient group.

Parameters	Malignant	Benign	*p*‐value
Number of patients, *n*	48	22	
Age, year	69.67 ± 7.64 (54–88)	66.36 ± 7.35 (52–78)	0.09
Total PSA, ng/mL	15.67 ± 9.56 (4.99–49.24)	10.88 ± 5.21 (4.33–20.05)	<0.05
fPSA/tPSA	0.11 ± 0.08 (0.01–0.33)	0.16 ± 0.05 (0.06–0.28)	<0.01
TRUS volume, mL	43.71 ± 20.09 (13–105)	66.14 ± 31.91 (25–147)	<0.01
PSA density, ng/mL^2^	0.49 ± 0.56 (0.04–1.17)	0.19 ± 0.14 (0.04–0.45)	0.02
Biopsy cores	11.73 ± 1.32 (8–13)	12	0.34
Positive biopsy cores	7.25 ± 3.69 (1–12)	0	<0.01
Lesion Gleason scores			
3 + 3 = 6	3	0	
3 + 4 = 7	6	0	
4 + 3 = 7	16	0	
4 + 4	13	0	
4 + 5	9	0	
5 + 5	1	0	
Pathological stage			
T1	0	0	
T2	34	0	
T3	13	0	
T4	1	0	
N0	0	0	
N1	3	0	
N2	0	0	
N3	0	0	

Abbreviations: fPSA, free PSA; PSA, prostate‐specific antigen; tPSA, total PSA; TRUS, transrectal ultrasound.

### 3.2 B‐model, CEUS imaging, and machine‐learning methods revealed 23 significant texture features

The importance score for the texture features was calculated, as shown in Figure S1A. In both the B‐mode and CEUS images, 368 radiomic texture features were filtered down to 23 significant texture features. These were used to build a classification model based on multiple logistic regression, a random forest model, and a support vector machine. For B‐mode images, when the number of features used to construct the classification model is greater than five, the accuracy of the classification model can exceed 0.95 or even be close to 1. For CEUS images, the accuracy of the classification model was close to 1 (Figure S1B–D).

### 3.3 Correlation analysis of texture and microvascular features identified 6 upregulated genes and 9 downregulated genes as markers of Pca progression

**FIGURE 2:**
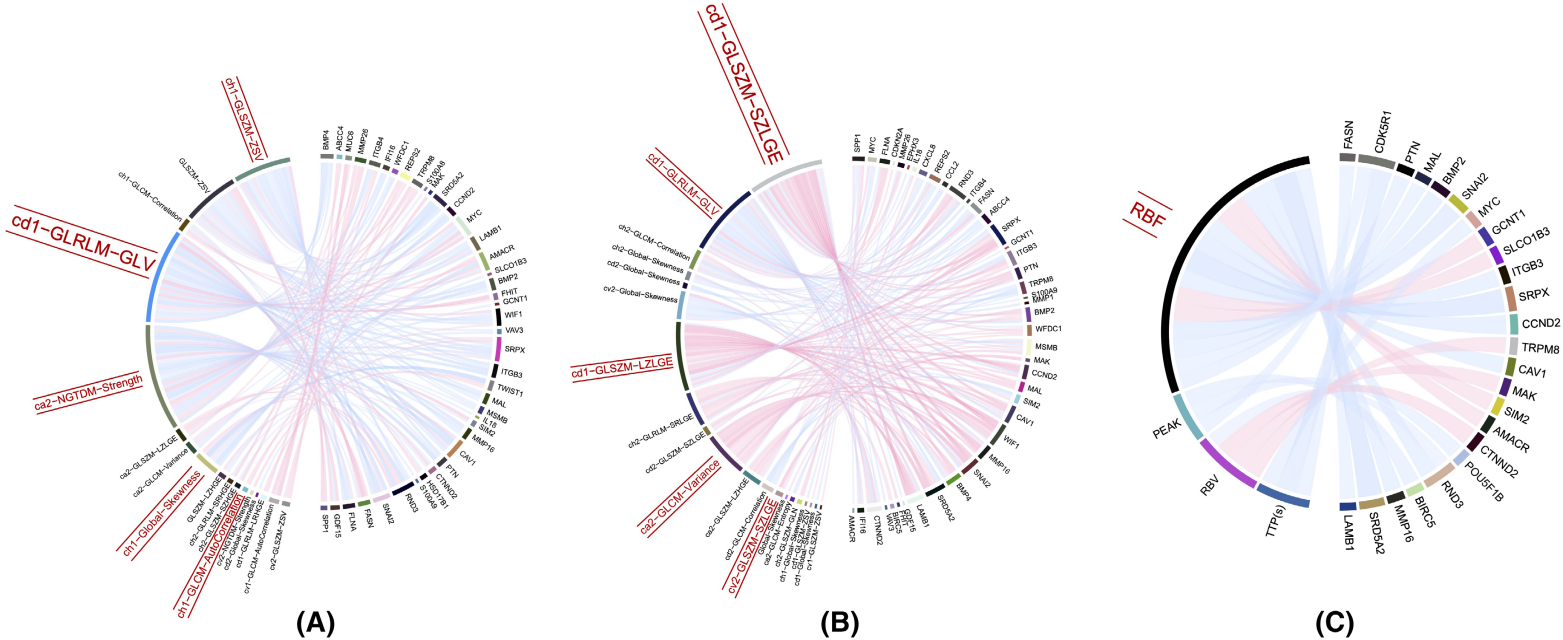
Summary of radiotranscriptomic correlations. (A) Colored circles refer to 23 significant texture features of B‐mode ultrasound (US) and genes; (B) Colored circles refer to 23 significant texture features of contrast‐enhanced ultrasound (CEUS) and genes; (C) Colored circles refer to 4 significant microvascular perfusion features of CEUS images and genes. The left half of the pie charts represents the features of radiomics, and the right counterpart represents the related genes. Ribbons connecting texture features and genes refer to significant correlations between them. Ribbon colors refer to the correlation coefficient sign (blue, negative; red, positive).

**FIGURE 3:**
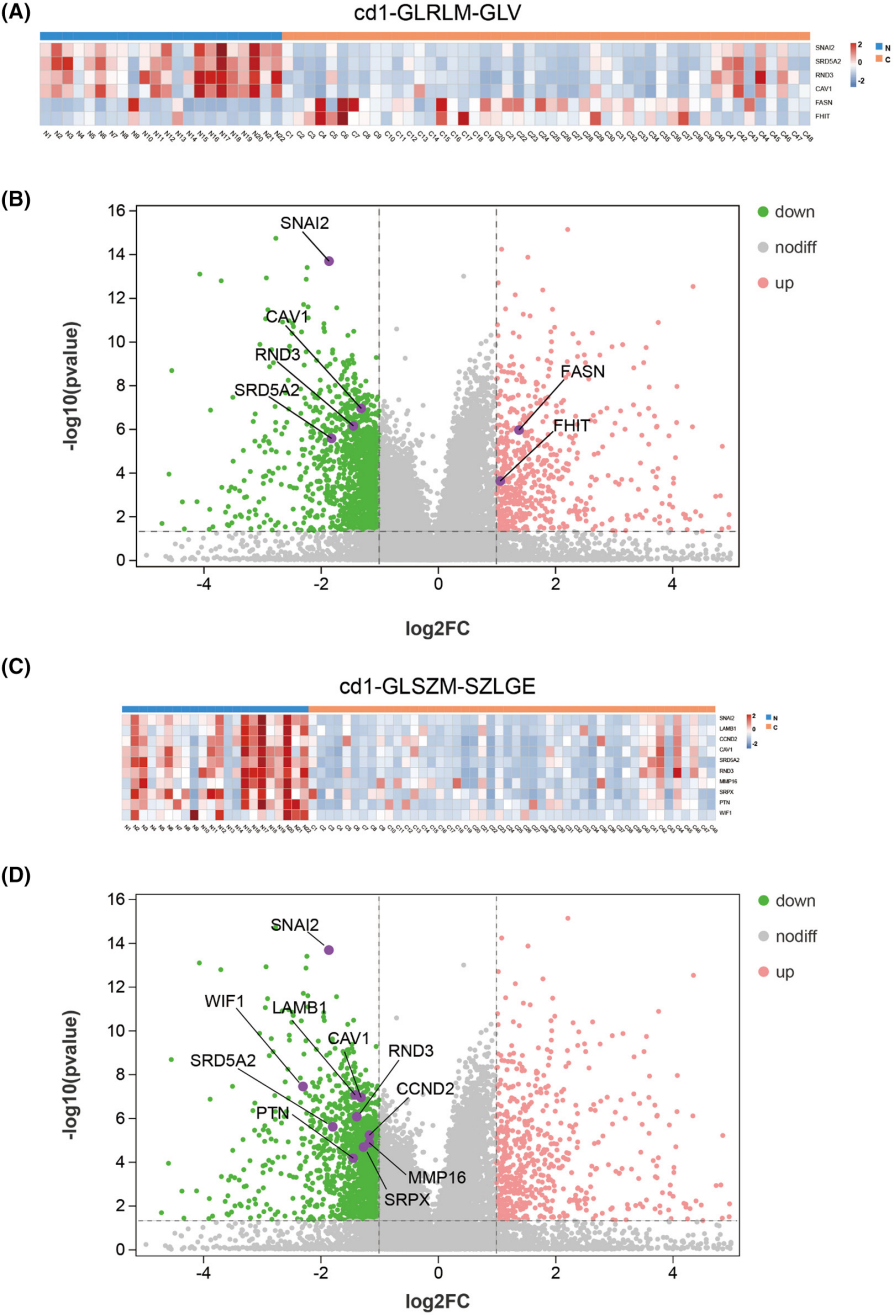
Radiotranscriptomic correlations according to key texture features with B‐mode ultrasound (US) and contrast‐enhanced ultrasound (CEUS). (A) A heat map image demonstrates six significant differentially expressed genes (DEGs) according to the cd1‐gray‐level route length matrix (GLRLM)‐gray‐level variance (GLV). The columns represent 70 individuals with either prostate cancer (PCa) (orange) or benign prostatic hyperplasia (BPH) (blue) and the rows represent six individuals with significant DEGs. The color key indicates the degree of DEGs in either direction: upregulation (orange) or downregulation (blue); (B) A volcano plot shows significant DEGs with cd1‐GLRLM‐GLV (*p* < 0.05). Purple circles represent significant DEGs between PCa and BPH with *p* < 0.05 and log_2_FC >2.0 or < −2.0; (C) A heat map shows 10 significant DEGs according to the cd1‐gray‐level size zone matrix (GLSZM)‐large zone low gray‐level emphasis (LZLGE). The columns represent 70 individuals with either PCa (orange) or BPH (blue), and the rows represent 10 individuals with significant DEGs. The color key indicates the degree of DEGs in either direction: upregulation (orange) or downregulation (blue); (D) A volcano plot demonstrates the significant DEGs with cd1‐GLSZM‐LZLGE (*p* < 0.05). Purple circles represent significant DEGs between PCa and BPH with a *p*‐value <0.05 and a log_2_FC >2.0 or < −2.0.

A total of 4080 DEGs were identified, including 1231 upregulated genes and 2849 downregulated genes, in PCa and BPH. In order to narrow the scope of the study, DO database was used for enrichment [DO (Disease Ontology), a database describing gene function and disease related], and 52 significantly DEGs were found to be related to PCa A total of 52 DEGs associated with PCa were identified. A full list of the 52 DEGs is presented in Table S2. To determine whether DEGs might be coupled based on radiomic texture features, we used pie‐chart mapping to understand all pairwise correlations between significant texture features, microvascular perfusion, and genes (Figure 2). Five significant texture features were highly correlated with different genes. DEGs associated with significant texture features and microvascular perfusion features are seen in Tables S3 and S4, respectively. Gene expression data according to the cd1‐gray‐level route length matrix (GLRLM)‐gray‐level variance (GLV) in B‐mode US using the heat map (Figure 3A) and volcano plots (Figure 3B) indicated that the cd1‐GLRLM‐GLV of PCa was associated with the upregulation of fatty acid synthase (FASN) and Fragile Histidine Triad (FHIT). This association is relevant to PCa in terms of lipid metabolism, androgen receptor status, drug resistance, proliferation, apoptosis, and the downregulation of steroid 5 alpha‐reductase 2 (SRD5A2), Snail family transcriptional repressor 2 (SNAI2), Rho family GTPase 3 (RND3), and caveolin‐1 (CAV1).^15^, ^16^ The latter are associated with PCa based on genes reported to be relevant in androgen receptor signaling inhibition, castration‐resistant PCa, and the proliferation and migration of PCa.^17^ Gene expression data according to cd1‐gray‐level size zone matrix (GLSZM)‐small zone low gray‐level emphasis (SZLGE) at CEUS using the heat map (Figure 3C) and volcano plots (Figure 3D) indicated that the cd1‐GLSZM‐large zone low gray‐level emphasis (LZLGE) is associated with the downregulation of WNT inhibitory factor 1 (W1F1), SNAI2, SRD5A2, Pleiotrophin (PTN), RND3, Laminin subunit beta 1 (LAMB1), Sushi repeat containing protein X‐linked (SRPX), CAV1, Matrix metallopeptidase 16 (MMP16), and Cyclin D2 (CCND2) genes, which are associated with the promotion of cell proliferation and metastasis based on the genes reported to be relevant to PCa.^18^

**FIGURE 4:**
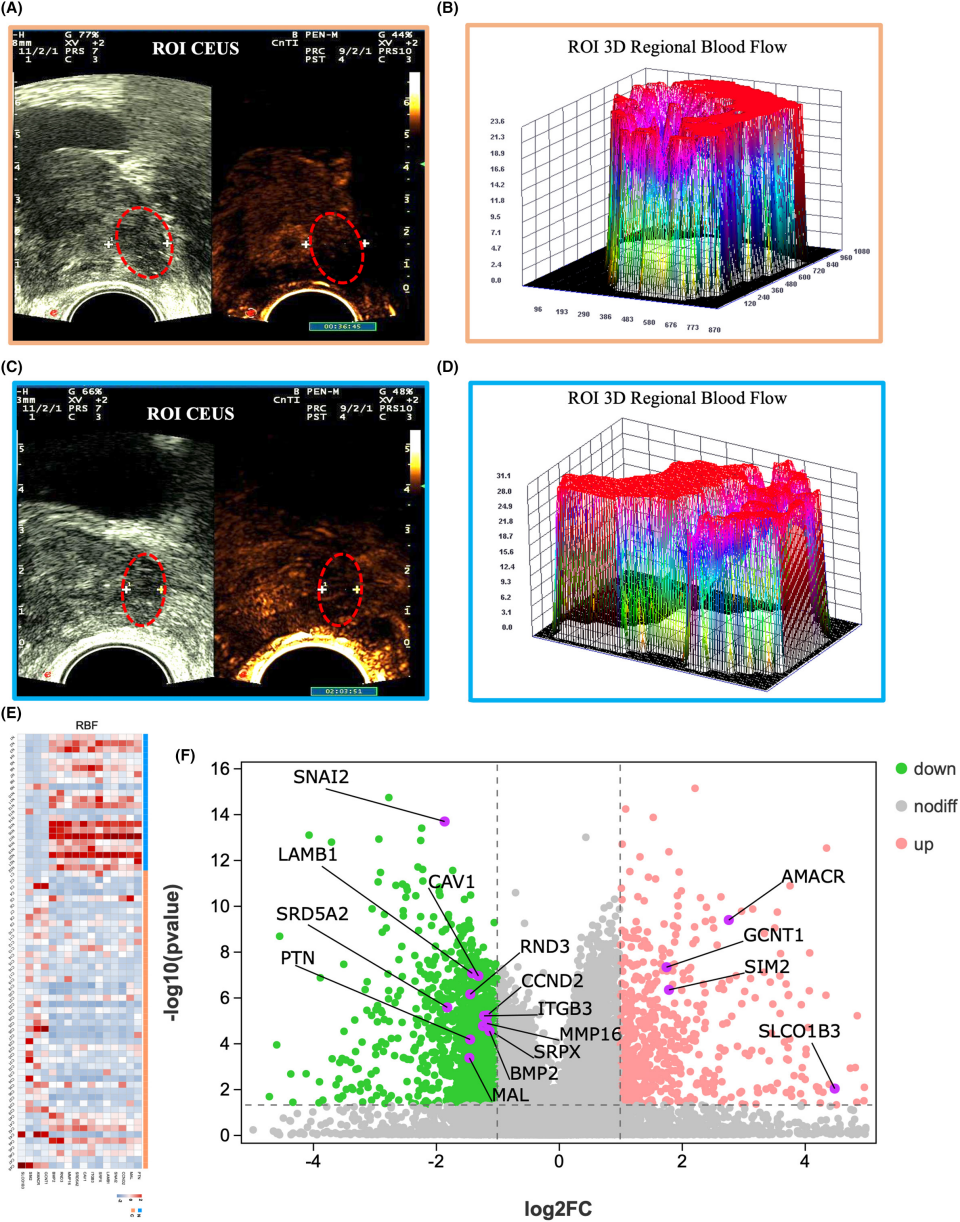
Radiotranscriptomic correlation according to the presence of microvascular perfusion in contrast‐enhanced ultrasound (CEUS) in 70 participants. (A) CEUS image (orange border) shows prostate cancer (PCa) in an 85‐year‐old man that does not penetrate the vessels; (B) CEUS image (blue border) shows benign prostatic hyperplasia (BPH) in a 79‐year‐old man with vessels that are partially penetrated (arrows). In the CEUS images, yellow circles are the regions of interest for measuring quantitative parameters; (C, D) Three‐dimensional images reveal enhancement with microvascular perfusion regional blood flow (RBF) in the region of interest of PCa and BPH tissues; (E) A heat map image demonstrates 16 DEGs according to the RBF. The columns represent 70 individuals with either PCa (orange) or BPH (blue), and the rows represent 16 individuals with DEGs. The color key indicates the degree of DEGs in either direction: upregulation (orange) or downregulation (blue); (F) A volcano plot shows 16 DEGs with RBF (*p* < 0.05). Purple circles represent DEGs between PCa and BPH with *p* < 0.05 and log_2_FC >2.0 or < −2.0.

Analysis of CEUS images revealed that PCa did not penetrate (Figure 4A) the vessels in an 85‐year‐old man, whereas BPH was found to partially penetrate (Figure 4B) the vessels in a 79‐year‐old man. Three‐dimensional images revealed an enhancement in microvascular perfusion and regional blood flow (RBF) in the ROI of PCa and BPH (Figure 4C,D). Gene expression data based on microvascular perfusion features using heat maps (Figure 4E) and volcano plots (Figure 4F) indicated that the RBF was associated with the upregulation of Solute carrier organic anion transporter family member 1B3 (SLCO1B3), Alpha‐methylacyl‐CoA racemase (AMACR), SIM2, Glucosaminyl (N‐acetyl) transferase 1 (GCNT1), PTN, and Catenin delta 2 (CTNND2) genes, which are relevant to androgen deprivation therapy resistance, early diagnosis biomarkers, tumor cell transcription factors, tumor aggressiveness, prometastasis, cell proliferation, and the migration and differentiation of PCa.^19^, ^20^, ^21^, ^22^, ^23^ They are also relevant to the downregulation of SNAI2, Mal, T‐cell differentiation protein (MAL), RND3, LAMB1, CAV1, MMP16, SRPX, ITGB3, and TGF‐beta signaling pathway (BMP2) genes, which are reported to be relevant to metastasis, cancer progression, motility, invasion into the surrounding extracellular matrix, and the proliferation, and migration of PCa.^24^, ^25^

### 3.4 53 and 63 miRNA gene networks, consisting of 9 and 14 mRNAs, were associated with the texture feature cd1‐GLSZM‐SZLGE and microvascular feature of PCa, respectively

**FIGURE 5:**
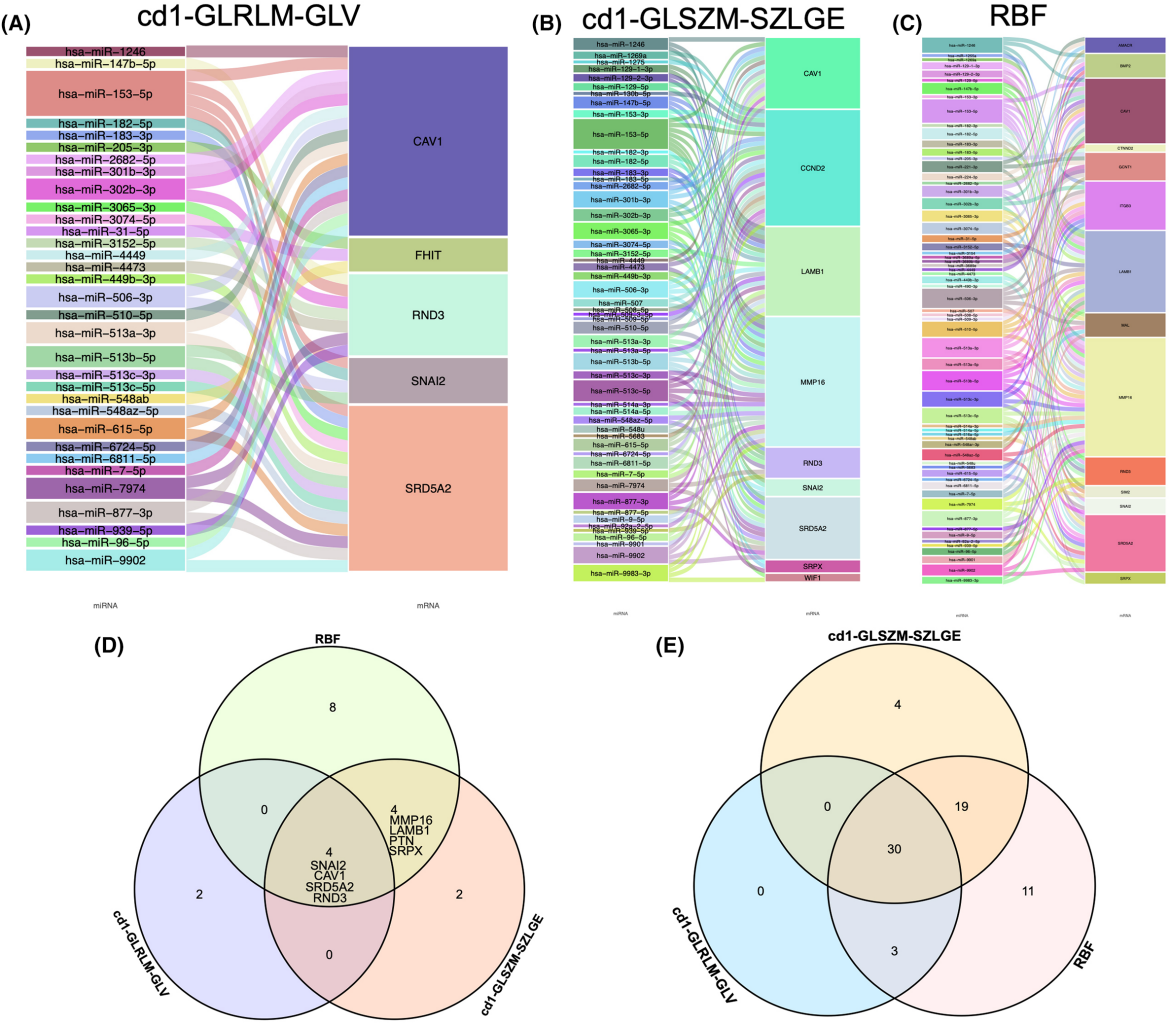
Sankey diagram for the co‐network of transcriptomics‐related mRNA and its co‐expressed microRNA (miRNA). (A) Core miRNA–gene network consisting of five mRNAs associated with the texture feature cd1‐gray‐level route length matrix (GLRLM)‐GLV of PCa and 33 miRNAs targeting the genes; (B) Core miRNA–gene network consisting of five mRNAs associated with texture feature cd1‐gray‐level size zone matrix (GLSZM)‐large zone low gray‐level emphasis (LZLGE) of PCa; (C) Core miRNA‐gene network consisting of 16 mRNAs associated with the microvascular feature RBF of PCa. The Venn diagram shows the mRNAs and miRNAs associated with three key features; (D) Four mRNA co‐expressions were obtained, including SNAI2, CAV1, RD5A2, and RND3; (E) Three miRNA co‐expressions were obtained, including miR205‐5p, miR205‐3p, and miR221‐3p.

In order to recognize the effect of radiotranscriptomics on miRNA‐mediated mRNAs in PCa, we built a regulatory network based on previously reported data. The correlation between the radiomic‐related features of the miRNA–mRNA regulatory network was analyzed using the Sankey diagram. As shown in Figure 5A–C, the core miRNA‐gene network comprised five mRNAs associated with the texture feature cd1‐GLRLM‐GLV of PCa and 33 miRNAs targeting these genes, among which two or more miRNAs might target the same mRNA. A total of 53 and 63 core miRNA gene networks, consisting of 9 and 14 mRNAs, were associated with the texture feature cd1‐GLSZM‐SZLGE and the microvascular features of PCa, respectively.

### 3.5 Seven coregulated mRNAs and miRNAs were associated with three key features, cd1‐GLRLM‐GLV, cd1‐GLSZM‐SZLGE, and RBF, respectively

The mRNAs and miRNAs associated with the three key features, cd1‐GLRLM‐GLV, cd1‐GLSZM‐SZLGE, and RBF, were mapped using a Venn diagram to identify the mRNAs and miRNAs shared by all three key features, as well as those shared by at least two key features. The following co‐regulated DEGs and differentially expressed miRNAs were found in the three datasets: SNAI2, CAV1, RD5A2, RND3, miR205‐5p, miR205‐3p, and miR221‐3p.

### 3.6 Pca pathways of differentially expressed genes were implicated in three key US imaging features

GO and KEGG pathway analyses were used to explore the enrichment of the functions and pathways of key features related to DEGs. The functions associated with PCa were annotated primarily with respect to the key features of the US images (Table S5). Significantly upregulated or downregulated genes with enriched functions were identified in three key US imaging features, including negative regulation of epithelial cell proliferation, androgen metabolic processes, male gonadal development, and lipid biosynthesis processes, which are associated with prostate tumorigenesis. Pathway enrichment analysis showed that key features related to the PCa pathways of DEGs (such as the Hippo signaling pathway and PCa) were implicated in three key US imaging features (Table S6).

### 3.7 New radiotranscriptomic analysis showed significant diagnostic advantages over traditional radiomic, transcriptomic, and clinical analyses alone

**FIGURE 6:**
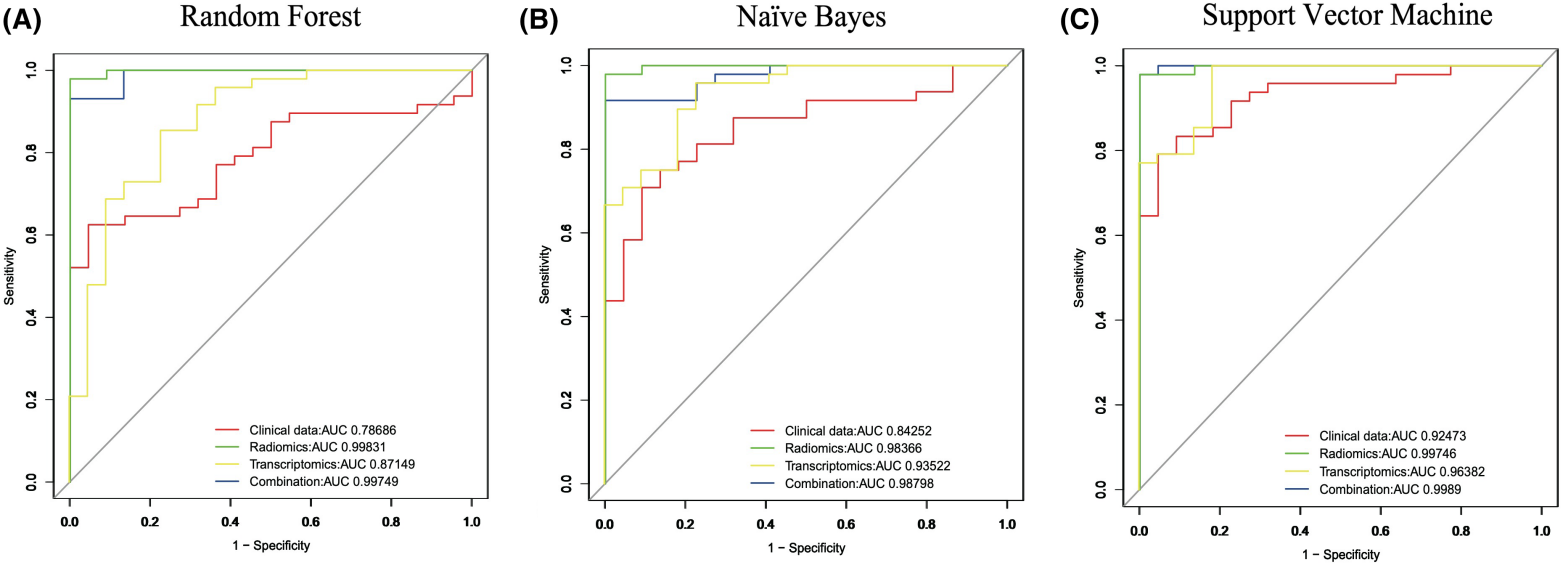
Prediction of performance using clinical features, transcriptomics, radiomics features, and combined features for predicting PCa. The receiver operating characteristic (ROC) curves of three machine‐learning methods (random forest, naïve Bayes, and SVM) to evaluate the dependability of the prediction accuracy of all the features in four feature sets: (A) Random forest mode; (B) Naive bayes mode; (C) Support vector machine mode. These curves were selected because their area under the curve value was closest to the average area value of the respective methods over all 10 runs (see also Table S7).

We used the receiver operating characteristic (ROC) curves of three machine‐learning methods (random forest, naïve Bayes, and SVM) to evaluate the dependability of the prediction accuracy of all the features in four feature sets (Figure 6): (1) the clinical dataset (age, PSA, free PSA/total PSA, volume, PSA denticity, and GS); (2) the molecular biomarkers (SNAI2, CAV1, RD5A2, RND3, miR205‐5p, miR205‐3p, and miR221‐3p); (3) the radiomics features only (texture features and microvascular perfusion features); and (4) the combined set. The values of the combined set (area under the curve [AUC] = 0.997, 0.987, and 0.998 for random forest, naïve Bayes, and SVM, respectively) showed more accurate (or equivalent) prognostic power than the radiomics feature (AUC = 0.998, 0.984, and 0.997), transcriptomics feature (genes and miRNAs) (AUC = 0.871, 0.935, and 0.064), or clinical datasets (AUC = 0.786, 0.843, and 0.925) (Table S7). These results highlighted the possibility of combining the features of different datasets to obtain a more accurate diagnosis of PCa.

## 4 DISCUSSION

TRUS plays an indispensable role in prostate biopsies. However, PCa is a multifocal and heterogeneous disease, which has a certain impact on TRUS‐guided prostate biopsy^26^ as 30% or more of prostate tumor specimens are isoechoic and the probability of cancer being recorded in hypoechoic lesions is approximately 50%. Our results included CEUS images indicating PCa that did not penetrate the vessels and BPH that partially penetrated the vessels. Tumor heterogeneity is difficult to assess by irregular sampling or biopsy. The establishment of a noninvasive technique to assess the heterogeneity of tumors is a clinical necessity. Texture analysis is an image processing method to quantify heterogeneity based on pixelwise spatial spread that may be missed by the naked eye. Imaging provides quantifiable, clinically relevant tumor parameters for the assessment of heterogeneity. The application of image signal heterogeneity and pattern recognition technology to the analysis of differences that cannot be detected by human eyes can greatly enhance and assist diagnosis. TRUS image texture features were used to distinguish normal, benign, and malignant prostate tissues.^27^ Among the different texture analysis methods, the wavelet transform and time‐frequency analysis techniques were the most prominent in terms of algorithm efficiency.^28^ We, therefore, analyzed 322 wavelet‐filtered features based on US images. Maggio et al. proposed a computer‐aided detection of PCa using discrete wavelet transform and texture features extracted from TRUS images; using texture biomarkers as an intratumoral heterogeneity quantification tool held great promise for the early detection of PCa.^29^, ^30^ We found that cd1‐GLRLM‐GLV was a common key texture feature for both B‐mode US and CEUS images and that GLSZM‐SZLGE was repeated many times in important CEUS texture features. Therefore, our results show that tumor heterogeneity can be quantified by radiologic imaging using texture heterogeneity features, which involves determining the spatial relationship of image voxel intensities within the region of interest. Therefore, texture features may be more suitable for detecting heterogeneity of PCa tissue structure in imaging.

Our experimental results demonstrated the importance of optimizing texture extraction parameters to improve their predictive value and better understand the relationship between texture and biology. Li et al. found that a radiomic‐based machine learning model could improve the prediction accuracy of clinically significant PCa compared with only evaluating the clinical risk factors associated with PCa.^31^ Sun et al. found that a horizontal comparison of texture features (gray‐level co‐occurrence matrix [GLCM] and gray‐level route length matrix [GLRLM]) improved the model performance for classifying prostate tumor aggressiveness.^32^

CEUS can reveal abnormal vascular areas of the prostate, thereby improving the number of positive biopsies performed.^33^ In a recently published study, perfusion‐ and dispersion‐related CEUS parameters were successfully combined using a machine learning approach.^34^ Our experimental results also confirmed that RBF was a key feature with the most DEGs among the four radiomic microvascular perfusion features. The latter characteristics can also act as strong indicators for PCa and provide insights into its underlying biology.

We observed strong individual correlations between PCa genes and quantitative radiomic features. The Gleason score classifies the degree of differentiation and growth pattern of the tumor into five grades; the higher the score, the worse the differentiation. Hence, Gleason score is closely related to prognosis, and is a tradeoff index for cell differentiation and invasion of PCa. FASN and FHIT are commonly upregulated during human PCa, and their inhibition was associated with promoting cell proliferation and invasion.^35^, ^36^ The *SNAI2* gene, which is silenced in PCa, regulates neuroendocrine differentiation, metastasis suppression, and expression of pluripotency genes.^37^, ^38^ Lower levels of expression of SNAI2 and CAV1 were associated with higher Gleason scores and advanced pathological T and N stages. Thus, SNAI2 and CAV1 can facilitate the identification of new molecules or pathways that may be involved in the diagnosis and treatment of PCa.^39^, ^40^ SRD5A2 is an important enzyme involved in androgen metabolism.^31^ RND3, which has been identified as a tumor suppressor gene, is constitutively expressed in BPH, whereas its expression is downregulated during prostate carcinogenesis.^41^, ^42^, ^43^ In agreement with this study, the PCa texture features was associated with the upregulation of FASN and FHIT genes, and the downregulation of SNAI2, SRD5A2, RND3, CAV1 genes. miRNA is a type of single‐stranded noncoding small RNA involved in posttranscriptional regulation of gene expression. Several lines of evidence have suggested that miRNAs play key roles in a variety of physiological events. Abnormal levels of miRNAs have been correlated with the occurrence and development of many diseases, including PCa. For instance, the expression of miR‐205 was significantly downregulated in advanced PCa.^44^ Studies have shown that the miR‐221 gene functions in disease pathogenesis by targeting a variety of cellular and molecular pathways.^45^

Functional analysis of the three key US imaging feature‐specific genes revealed diverse functional GO terms and KEGG pathways. Significantly upregulated or downregulated genes in three key US imaging features were enriched for functions including negative regulation of epithelial cell proliferation, androgen metabolic processes, male gonadal development, and lipid biosynthesis processes associated with prostate tumorigenesis. More gene‐related PCa pathways (such as the Hippo signaling pathway and PCa) were involved in the three key US imaging characteristics.

Recent studies have used other machine learning models, such as neurofuzzy models (AUC = 0.812)^46^ and artificial networks (AUC = 0.695),^47^ to predict PCa staging based solely on clinical characteristics. These AUC values were similar to those calculated by our machine learning methods using clinical characteristics (0.786, 0.843, and 0.925, random forest, naïve Bayes, and SVM, respectively). Our combined set prediction model showed high potential to yield clinically relevant results for characterizing PCa (AUC = 0.997, 0.987, and 0.998, for random forest, naïve Bayes, and SVM, respectively).

Our study had several limitations. First, limiting the occurrence of false‐positives in the application of radiomics is important. This study uses a variety of methods to minimize the occurrence of false positives. We acknowledge that although radiotranscriptomic analysis is highly innovative, it is also a truly arduous undertaking as it is still in its early stages of development. Future studies are needed to validate these findings using a larger cohort of patients. Second, PC diagnosis was made based on clinical findings, US imaging findings, pathological analysis, and transcriptomic results. We used the ROC curves to evaluate the dependability of the prediction accuracy of radiotranscriptomics; however, future analysis is needed to determine whether radiotranscriptomic analysis can improve the sensitivity and specificity of PCa diagnosis with US imaging. Third, radiomics features are calculated from matrices that describe various spatial relationships between signal intensities of voxels. Voxel size resampling is required as a preprocessing step for datasets acquired with variable voxel sizes. In future In future 3D (volume)ultrasound imaging radiomics studies, we will normalize the size‐dependent features by the number of voxels to reduce the dependence on the number of voxels in the ROI. Finally, the Gleason score is closely related to prognosis and a tradeoff index for cell differentiation and invasion of PCa. Our next research goal is to combine stage‐specific genes and miRNAs with aggressiveness‐related imaging features, thereby constructing radiotranscriptomic signatures for improving the prediction accuracy for stages T2 and T3 in patients with PCa.

## 5 CONCLUSIONS

In conclusion, when combining traditional clinical features and using the power of radiotranscriptomics, the identified biomarkers (SNAI2, CAV1, RD5A2, RND3, miR205‐5p, miR205‐3p, and miR221‐3p) could improve the accuracy of predicting PCa. This approach can provide unprecedented opportunities to improve and support medical decision making, given the routine use of imaging in diagnosis and treatment in clinical practice worldwide.

## AUTHOR CONTRIBUTIONS

**Qian Yang:** Conceptualization (lead); data curation (lead); formal analysis (lead); funding acquisition (lead); investigation (lead); methodology (lead); resources (lead); writing – original draft (lead); writing – review and editing (lead). **Qiuyang Li:** Conceptualization (equal); resources (equal). **Nan Li:** Data curation (lead); software (equal). **Dingyi Wang:** Data curation (equal); formal analysis (equal); validation (equal). **Shaoxi Niu:** Data curation (equal); software (equal); validation (equal). **Tang Peng:** Formal analysis (equal); investigation (equal); software (equal). **Jing Xiao:** Data curation (equal); resources (equal). **Jiahang Zhao:** Data curation (equal); resources (equal). **Pei Wang:** Data curation (equal); resources (equal). **Yukun Luo:** Project administration (lead); supervision (equal). **Jie Tang:** Project administration (lead); supervision (equal).

## FUNDING INFORMATION

This work was supported by grants from the National Natural Science Foundation of China (nos. 81471682 and 81801708), China Postdoctoral Science Foundation (no. 2021T140795), Natural Science Basic Research Program of Shaanxi Province (no.2023‐JC‐QN‐0912), Xi'an Science and Technology Plan Project (no. 21YXYJ0134), and Xi'an International Medical Center Hospital Level Project (no. 2022QN01).

## CONFLICT OF INTEREST STATEMENT

The authors declare that the research was conducted in the absence of any specific financial interests, relationships, or affiliations relevant to the subject matter or materials discussed in the manuscript (e.g., employment/affiliation; grants or funding; consultancies; honoraria; stock ownership or options; expert testimony; royalties; or patents filed, received, or pending) that could be construed as potential conflicts of interest.

## Supporting information


Data S1.

## Data Availability

The data presented in this study are available on request from the corresponding authors (J.T. and Y.L.). The data are not publicly available owing to hospital regulations. This study was conducted in accordance with the Declaration of Helsinki and approved by the First Center of the Chinese PLA General Hospital (S2021‐565‐01). The requirement for patient consent was waived by the ethics committees owing to the retrospective nature of the study.
