# Supplementary material for: Radiotranscriptomics identified new mRNAs and miRNA markers for distinguishing prostate cancer from benign prostatic hyperplasia
Source: Cancer Med. 2023 Nov 21;12(24):21694–708. doi: 10.1002/cam4.6728 (PMC10757143; doi:10.1002/cam4.6728)
Supplement: Supplementary file 1 — Data S1. [file CAM4-12-21694-s001.docx]

**Supporting Information**

Radiotranscriptomics identified new mRNAs and miRNA markers for distinguishing prostate cancer from benign prostatic hyperplasia

**Radiomics Texture Features**

***Extraction of texture features.*** We identified 368 candidates with radiomic texture features that described the tumor characteristics of the region of interest (ROI) from B-mode and CEUS images. Texture features were divided into three groups: (1) global features, (2) higher-order texture features, and (3) wavelet features. The first group comprised six global features that were quantified. Group 2 included 40 features extracted from the gray-level size zone matrix (GLSZM), gray-level route length matrix (GLRLM), gray-level co-occurrence matrix (GLCM), and neighborhood gray tone difference matrix. Group 3, comprising 322 wavelet-filtered features, was investigated. For the description of the name of the wavelet feature, “cv1” is the vertical image of the first-order wavelet decomposition; “cv2” is the vertical image of the second-order wavelet decomposition; “ch1” is the horizontal image of the first order wavelet decomposition; “ch2” is the horizontal image of the second-order wavelet decomposition; “ch1” is the 45° image of the first order wavelet decomposition; “cd” is the 45° direction image of the second order; and “ca2” is the image sampled twice under wavelet decomposition. The authors will provide additional information on how the texture features align with the Image Biomarker Standardisation Initiative (IBSI) guidelines, ensuring reproducibility and standardization.

***Selection of texture features.*** A stepwise feature selection method was used to screen out the features most relevant to disease classification but least relevant between features. First, the feature that was most closely correlated with the disease classification variables was selected as the priming feature. The feature most related to the disease classification variables but least related to the priming features was selected as the second feature. The average of the correlation coefficients of this feature with all retained features, plus the correlation coefficients of this feature with categorical variables, was used as the score for this feature. When the score of a feature dropped sharply, the stepwise feature selection procedure was terminated. Multivariate logistic regression, support vector machine (SVM), and random forest were used to train the disease classification models. A stepwise feature-selection method was used to select the most effective feature combination for the disease classification model. In the training process of the model, an unbalanced data sampling method was adopted; that is, random sampling was conducted 100 times. In each random sampling process, approximately 30% of the samples were selected as the test set for model evaluation. These samples will not be included in the training set. The features that occurred in combinations with a classification performance greater than 0.98 were counted. A full list of the frequencies of the features under different evaluation models is presented in Table S1.

***Microvascular perfusion features.*** QontraX software was used to process the perfusion in each of the previously determined ROIs, automatically calculate the parameters, and plot the measured and calculated curves. Microvascular perfusion features included peak% (the maximum signal intensity reached during the SonoVue bolus transit at time T in the selected ROI during the selected period of enhancement), time to peak (in seconds, the time from the onset of tumoral enhancement to reaching the maximum signal intensity), regional blood volume (mm^3^, the volume of blood in the ROI proportional to the area under the time-intensity curve), and regional blood flow (RBF) (mm^3^/s, the regional blood volume-to-main transit time ratio).


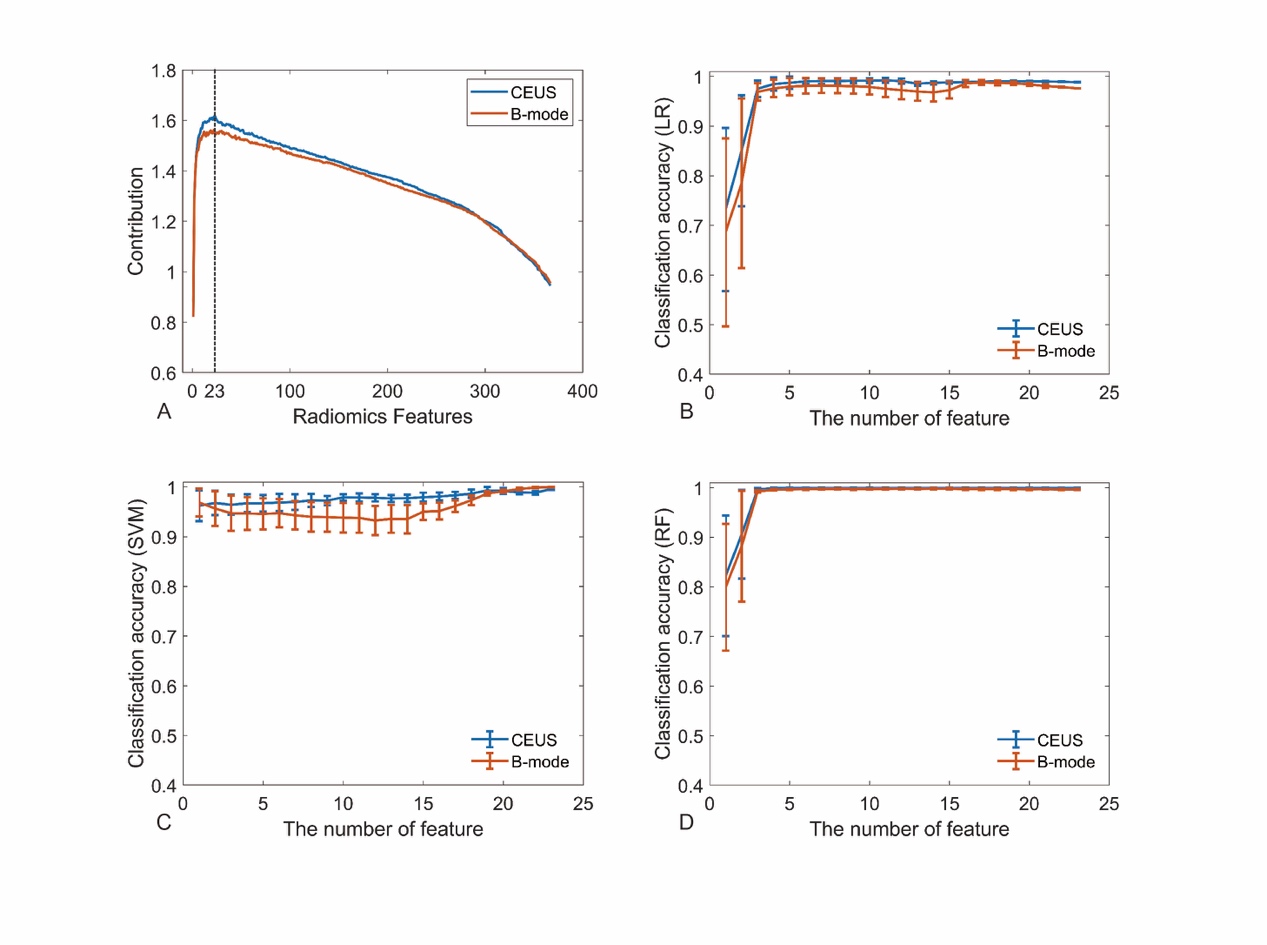


**Figure S1.** Evaluation models of prostate cancer based on radiomics features. **(A)** The important scores of the features. In both the B-mode and CEUS images, the 23 radiomic features were used to construct a classification model. CEUS = contrast-enhanced ultrasound; **(B)** LR = logistic regression mode; **(C)** SVM = support vector machine mode; **(D)** RF = random forest mode.

**Table S1.** (a) Frequency of features under different evaluation models: gray image

| **Multivariate logistic regression** | | **Random forest** | | **Support vector machine** | |
| --- | --- | --- | --- | --- | --- |
| **Radiomic features** | **Frequency of features** | **Radiomic features** | **Frequency of features** | **Radiomic features** | **Frequency of features** |
| 'ca2-GLSZM-LZLGE' | 3120 | 'ch1-GLSZM-ZSV' | 5338 | 'cd2-GLRLM-LGRE' | 167 |
| 'cd1-GLRLM-LRHGE' | 2502 | 'ca2-NGTDM-Strength' | 4688 | 'ch1-Global-Skewness' | 165 |
| 'cd1-GLSZM-ZSV' | 2398 | 'cd1-GLRLM-LRHGE' | 3138 | 'ch2-Global-Skewness' | 164 |
| 'ca2-NGTDM-Strength' | 1956 | 'GLSZM-LZHGE' | 2924 | 'ch1-GLCM-AutoCorrelation' | 158 |
| 'cv1-GLCM-AutoCorrelation' | 1857 | 'ca2-GLCM-Variance' | 2875 | 'ch1-GLCM-Correlation' | 152 |
| 'ch2-GLRLM-SRHGE' | 1823 | 'cv2-NGTDM-Strength' | 2837 | 'cv2-NGTDM-Strength' | 152 |
| 'ch2-GLSZM-SZHGE' | 1604 | 'cd1-GLSZM-ZSV' | 2526 | 'cd1-GLSZM-ZSN' | 150 |
| 'cv2-NGTDM-Strength' | 1574 | 'cd1-Global-Skewness' | 2497 | 'cd1-Global-Skewness' | 146 |
| 'ch1-Global-Skewness' | 1423 | 'cd2-Global-Skewness' | 2457 | 'cd1-GLSZM-ZSV' | 145 |
| 'cv2-GLSZM-ZSV' | 1393 | 'cv1-GLCM-AutoCorrelation' | 2327 | 'ch2-GLRLM-SRHGE' | 142 |
| 'cd1-Global-Skewness' | 1308 | 'ch2-GLSZM-SZHGE' | 2059 | 'cv1-GLCM-AutoCorrelation' | 141 |
| 'ch2-Global-Skewness' | 1270 | 'ch1-GLCM-AutoCorrelation' | 2023 | 'ca2-GLCM-Variance' | 139 |
| 'ch1-GLCM-AutoCorrelation' | 1221 | 'cd1-GLSZM-ZSN' | 2021 | 'ch2-GLSZM-SZHGE' | 139 |
| 'cd2-Global-Skewness' | 1168 | 'cd2-GLRLM-LGRE' | 1956 | 'GLSZM-LZHGE' | 138 |
| 'cd2-GLRLM-LGRE' | 1155 | 'ca2-GLSZM-LZLGE' | 1930 | 'cd2-Global-Skewness' | 126 |
| 'cd1-GLRLM-GLV' | 881 | 'ca2-GLSZM-LZLGE' | 1909 | 'cv2-GLSZM-ZSV' | 125 |
| 'GLSZM-LZHGE' | 841 | 'ch1-GLCM-Correlation' | 1656 | 'cd1-GLRLM-LRHGE' | 123 |
| 'cd1-GLSZM-ZSN' | 773 | 'ch2-GLRLM-SRHGE' | 1650 | 'GLRLM-GLV' | 120 |
| 'GLSZM-ZSV' | 742 | 'ch1-Global-Skewness' | 1407 | 'ca2-GLSZM-LZLGE' | 113 |
| 'ch1-GLSZM-ZSV' | 525 | 'GLSZM-ZSV' | 1136 | 'GLSZM-ZSV' | 108 |
| 'ca2-GLCM-Variance' | 503 | 'GLRLM-GLV' | 937 | 'cd1-GLRLM-GLV' | 76 |
| 'GLRLM-GLV' | 497 | 'cv2-GLSZM-ZSV' | 747 | 'ch1-GLSZM-ZSV' | 75 |
| 'ch1-GLCM-Correlation' | 380 | 'cd1-GLRLM-GLV' | 739 | 'ca2-NGTDM-Strength' | 35 |

**Table S1.** (b) Frequency of features under different evaluation models: color image

| **Multivariate logistic regression** | | **Random forest** | | **Support vector machine** | |
| --- | --- | --- | --- | --- | --- |
| **Radiomic features** | **Frequency of features** | **Radiomic features** | **Frequency of features** | **Radiomic features** | **Frequency of features** |
| 'ch1-Global-Skewness' | 6152 | 'cv1-GLSZM-ZSV' | 6924 | 'cd2-Global-Skewness' | 259 |
| 'ch1-GLCM-Correlation' | 4429 | 'ca2-Global-Kurtosis' | 4803 | 'ca2-Global-Kurtosis' | 245 |
| 'ca2-Global-Kurtosis' | 4178 | 'cd1-Global-Skewness' | 4548 | 'ch1-Global-Skewness' | 239 |
| 'Global-Skewness' | 4067 | 'cv2-GLSZM-SZLGE' | 4172 | 'ch2-Global-Skewness' | 238 |
| 'cd2-Global-Skewness' | 3649 | 'cd1-GLSZM-GLV' | 3609 | 'ca2-GLCM-Entropy' | 235 |
| 'ch2-GLSZM-GLN' | 3570 | 'Global-Skewness' | 3370 | 'ch2-GLSZM-GLN' | 223 |
| 'cd2-GLSZM-SZLGE' | 3520 | 'ch2-Global-Skewness' | 3233 | 'cd1-Global-Skewness' | 214 |
| 'cd2-GLCM-Correlation' | 3254 | 'ch2-GLSZM-GLN' | 3213 | 'ca2-Global-Skewness' | 212 |
| 'cv1-GLSZM-ZSV' | 3111 | 'ca2-GLCM-Entropy' | 3116 | 'cv1-GLSZM-ZSV' | 212 |
| 'ch2-GLCM-Correlation' | 3098 | 'cd2-GLCM-Correlation' | 3102 | 'Global-Skewness' | 203 |
| 'cv2-Global-Skewness' | 3004 | 'cd2-GLSZM-SZLGE' | 2758 | 'cd2-GLSZM-SZLGE' | 200 |
| 'cv2-GLSZM-SZLGE' | 3002 | 'ch2-GLRLM-SRLGE' | 2629 | 'ch2-GLRLM-SRLGE' | 197 |
| 'cd1-GLSZM-ZSV' | 2597 | 'cd2-Global-Skewness' | 2583 | 'ch1-GLCM-Correlation' | 193 |
| 'ch2-GLRLM-SRLGE' | 2357 | 'cd1-GLSZM-ZSV' | 2475 | 'cd1-GLSZM-ZSV' | 191 |
| 'ca2-GLSZM-LZHGE' | 2242 | 'ch1-GLCM-Correlation' | 2295 | 'cv2-GLSZM-SZLGE' | 185 |
| 'cd1-GLSZM-SZLGE' | 2092 | 'cd1-GLSZM-SZLGE' | 2218 | 'cv2-Global-Skewness' | 184 |
| 'ca2-Global-Skewness' | 1907 | 'ca2-Global-Skewness' | 2185 | 'ca2-GLSZM-LZHGE' | 181 |
| 'cd1-GLSZM-GLV' | 1821 | 'ch2-GLCM-Correlation' | 2185 | 'cd2-GLCM-Correlation' | 147 |
| 'ch2-Global-Skewness' | 1460 | 'cv2-Global-Skewness' | 2162 | 'cd1-GLSZM-SZLGE' | 120 |
| 'ca2-GLCM-Variance' | 1390 | 'ca2-GLCM-Variance' | 1918 | 'cd1-GLSZM-GLV' | 95 |
| 'ca2-GLCM-Entropy' | 1316 | 'ch1-Global-Skewness' | 1824 | 'ch2-GLCM-Correlation' | 93 |
| 'cd1-Global-Skewness' | 1229 | 'cd1-GLSZM-LZLGE' | 1595 | 'cd1-GLSZM-LZLGE' | 71 |
| 'cd1-GLSZM-LZLGE' | 1209 | 'ca2-GLSZM-LZHGE' | 1504 | 'ca2-GLCM-Variance' | 40 |

**Table S2.** The list of 52 differentially expressed genes related to the prostate cancer.

| **ID** | **Symbol** | **log_2_(fc)** | ***P*-value** | **FDR** |
| --- | --- | --- | --- | --- |
| ENSG00000019549 | SNAI2 | −1.85571 | 2.12498E-14 | 4.58217E-11 |
| ENSG00000089685 | BIRC5 | 1.600794 | 4.33695E-05 | 0.000452511 |
| ENSG00000091136 | LAMB1 | −1.40692 | 8.97175E-08 | 5.32461E-06 |
| ENSG00000101955 | SRPX | −1.23143 | 1.78644E-05 | 0.000230976 |
| ENSG00000103175 | WFDC1 | −1.06498 | 0.00071113 | 0.003941753 |
| ENSG00000105131 | EPHX3 | −1.04063 | 0.019363733 | 0.052943358 |
| ENSG00000105894 | PTN | −1.44066 | 6.9183E-05 | 0.000654305 |
| ENSG00000105974 | CAV1 | −1.30677 | 1.16674E-07 | 6.41444E-06 |
| ENSG00000108691 | CCL2 | −1.11687 | 0.000269246 | 0.001872168 |
| ENSG00000108786 | HSD17B1 | 1.017403 | 4.74323E-06 | 8.87675E-05 |
| ENSG00000111700 | SLCO1B3 | 4.489179 | 0.009598075 | 0.030436247 |
| ENSG00000111837 | MAK | 1.397886 | 2.33397E-05 | 0.00028031 |
| ENSG00000115963 | RND3 | −1.43906 | 7.35069E-07 | 2.31959E-05 |
| ENSG00000118785 | SPP1 | 1.150811 | 0.002569649 | 0.0107361 |
| ENSG00000118971 | CCND2 | −1.16319 | 6.12316E-06 | 0.000107492 |
| ENSG00000122691 | TWIST1 | 2.973901 | 1.75981E-10 | 5.6921E-08 |
| ENSG00000125257 | ABCC4 | 1.113064 | 2.87726E-05 | 0.000330213 |
| ENSG00000125378 | BMP4 | −1.15166 | 3.91625E-06 | 7.67039E-05 |
| ENSG00000125845 | BMP2 | −1.125 | 2.98307E-05 | 0.000337959 |
| ENSG00000127533 | F2RL3 | 1.720461 | 2.00428E-05 | 0.000249531 |
| ENSG00000130513 | GDF15 | 2.305894 | 4.51803E-11 | 1.86556E-08 |
| ENSG00000132470 | ITGB4 | −1.01861 | 0.000474659 | 0.002864334 |
| ENSG00000134215 | VAV3 | −1.10497 | 0.000445932 | 0.002740405 |
| ENSG00000136997 | MYC | 1.958266 | 3.40242E-12 | 2.75128E-09 |
| ENSG00000143546 | S100A8 | −1.47137 | 3.30932E-05 | 0.000365532 |
| ENSG00000144481 | TRPM8 | 1.236252 | 0.000176697 | 0.00135887 |
| ENSG00000147889 | CDKN2A | 1.061624 | 0.029431033 | 0.074187304 |
| ENSG00000150782 | IL18 | −1.03867 | 0.000711548 | 0.003941753 |
| ENSG00000156076 | WIF1 | −2.24805 | 7.19279E-08 | 4.53216E-06 |
| ENSG00000156103 | MMP16 | −1.1663 | 1.37616E-05 | 0.000190493 |
| ENSG00000159263 | SIM2 | 1.793866 | 4.71974E-07 | 1.68066E-05 |
| ENSG00000160182 | TFF1 | 1.007362 | 0.007750457 | 0.025782159 |
| ENSG00000163220 | S100A9 | −1.57749 | 0.001250021 | 0.006090672 |
| ENSG00000163565 | IFI16 | −1.18291 | 5.64684E-05 | 0.000559123 |
| ENSG00000167346 | MMP26 | 2.777624 | 1.7201E-07 | 8.26286E-06 |
| ENSG00000169429 | CXCL8 | −1.96751 | 1.0651E-05 | 0.000159617 |
| ENSG00000169710 | FASN | 1.386713 | 1.13917E-06 | 3.19016E-05 |
| ENSG00000169862 | CTNND2 | 1.180985 | 6.22422E-07 | 2.04043E-05 |
| ENSG00000169891 | REPS2 | 1.24003 | 3.87136E-08 | 2.88967E-06 |
| ENSG00000172005 | MAL | −1.45331 | 0.000441194 | 0.002716448 |
| ENSG00000176749 | CDK5R1 | 1.496289 | 4.6609E-07 | 1.66768E-05 |
| ENSG00000184956 | MUC6 | 3.698898 | 2.62447E-07 | 1.09065E-05 |
| ENSG00000187210 | GCNT1 | 1.754001 | 4.84675E-08 | 3.34736E-06 |
| ENSG00000189283 | FHIT | 1.066034 | 0.000245871 | 0.001752337 |
| ENSG00000196611 | MMP1 | −4.58725 | 0.000118675 | 0.000991872 |
| ENSG00000196924 | FLNA | −1.05701 | 0.000251802 | 0.001782822 |
| ENSG00000198788 | MUC2 | 1.837426 | 0.001577742 | 0.007331569 |
| ENSG00000212993 | POU5F1B | 1.096695 | 6.69931E-05 | 0.000637633 |
| ENSG00000242110 | AMACR | 2.766662 | 4.22596E-10 | 1.13907E-07 |
| ENSG00000259207 | ITGB3 | −1.21447 | 6.41724E-06 | 0.00011069 |
| ENSG00000263639 | MSMB | −1.65774 | 0.005754441 | 0.020293884 |
| ENSG00000277893 | SRD5A2 | −1.81266 | 2.75634E-06 | 5.94123E-05 |

**Table S3.** Differentially expressed genes relevant to prostate cancer according to the ultrasound texture features

| **US phenotype** | **Gene name** | **Log2FC** | **r** | ***P*-value** |
| --- | --- | --- | --- | --- |
| **B-mode US** |  |  |  |  |
| **cd1-GLRLM-GLV** |  |  |  |  |
| FASN | Fatty acid synthase | 1.39 | 0.46 | *P* < 0.01 |
| FHIT | Fragile histidine triad diadenosine triphosphatase | 1.07 | 0.44 | *P* < 0.01 |
| SNAI2 | Snail family transcriptional repressor 2 | −1.86 | −0.41 | *P* < 0.01 |
| RD5A2 | Steroid 5 alpha-reductase 2 | −1.81 | −0.41 | *P* < 0.01 |
| RND3 | Rho family GTPase 3 | −1.44 | −0.43 | *P* < 0.01 |
| CAV1 | Caveolin 1 | −1.31 | −0.44 | *P* < 0.01 |
| **ca2-NGTDM-Strength** |  |  |  |  |
| SNAI2 | Snail family transcriptional repressor 2 | −1.86 | −0.41 | *P* < 0.01 |
| SRD5A2 | Steroid 5 alpha-reductase 2 | −1.81 | −0.43 | *P* < 0.01 |
| RND3 | Rho family GTPase 3 | −1.44 | −0.45 | *P* < 0.01 |
| CAV1 | Caveolin 1 | −1.31 | −0.46 | *P* < 0.01 |
| **ch1-Global-Skewness** |  |  |  |  |
| TWIST1 | Twist family bHLH transcription factor 1 | 2.97 | −0.53 | *P* < 0.01 |
| AMACR | Alpha-methylacyl-CoA racemase | 2.77 | −0.41 | *P* < 0.01 |
| **cv1-GLCM-Auto Correlation** |  |  |  |  |
| MUC6 | Mucin 6 | 3.69 | 0.42 | *P* < 0.01 |
| **ch1-GLSZM-ZSV** |  |  |  |  |
| SRD5A2 | Steroid 5 alpha-reductase 2 | −1.81 | −0.40 | *P* < 0.01 |
| **CEUS** |  |  |  |  |
| **cd1-GLSZM-SZLGE** |  |  |  |  |
| WIF1 | WNT inhibitory factor 1 | −2.25 | 0.56 | *P* < 0.01 |
| SNAI2 | Snail family transcriptional repressor 2 | −1.86 | 0.55 | *P* < 0.01 |
| SRD5A2 | Steroid 5 alpha-reductase 2 | −1.81 | 0.52 | *P* < 0.01 |
| PTN | Pleiotrophin | −1.44 | 0.41 | *P* < 0.01 |
| RND3 | Rho family GTPase 3 | −1.43 | 0.42 | *P* < 0.01 |
| LAMB1 | Laminin subunit beta 1 | −1.41 | 0.56 | *P* < 0.01 |
| CAV1 | Caveolin-1 | −1.31 | 0.45 | *P* < 0.01 |
| SRPX | Sushi repeat containing protein X-linked | −1.23 | 0.51 | *P* < 0.01 |
| MMP16 | Matrix metallopeptidase 16 | −1.17 | 0.51 | *P* < 0.01 |
| CCND2 | Cyclin D2 | −1.16 | 0.46 | *P* < 0.01 |
| **cd1-GLSZM-LZLGE** |  |  |  |  |
| WIF1 | WNT inhibitory factor 1 | −2.25 | 0.54 | *P* < 0.01 |
| SNAI2 | Snail family transcriptional repressor 2 | −1.86 | 0.53 | *P* < 0.01 |
| SRD5A2 | Steroid 5 alpha-reductase 2 | −1.81 | 0.54 | *P* < 0.01 |
| LAMB1 | Laminin subunit beta 1 | −1.41 | 0.52 | *P* < 0.01 |
| SRPX | Sushi repeat containing protein X-linked | −1.23 | 0.55 | *P* < 0.01 |
| CAV1 | Caveolin-1 | −1.31 | 0.44 | *P* < 0.01 |
| MMP16 | Matrix metallopeptidase 16 | −1.17 | 0.49 | *P* < 0.01 |
| CCND2 | Cyclin D2 | −1.16 | 0.48 | *P* < 0.01 |
| **ca2-GLCM-Variance** |  |  |  |  |
| W1F1 | WNT inhibitory factor 1 | 2.25 | 0.52 | *P* < 0.01 |
| SNAI2 | Snail family transcriptional repressor 2 | −1.86 | 0.46 | *P* < 0.01 |
| PTN | Pleiotrophin | −1.44 | 0.40 | *P* < 0.01 |
| LAMB1 | Laminin subunit beta 1 | −1.41 | 0.42 | *P* < 0.01 |
| **cd1-GLRLM-GLV** |  |  |  |  |
| FASN | Fatty acid synthase | 1.39 | 0.42 | *P* < 0.01 |
| RND3 | Rho family GTPase 3 | −1.44 | −0.42 | *P* < 0.01 |
| **cv2-GLSZM-SZLGE** |  |  |  |  |
| MAK | Male germ cell cell-associated kinase | 1.39 | 0.45 | *P* < 0.01 |

**Table S4.** Differentially expressed genes relevant to prostate cancer according to the microvascular perfusion phenotypes

| **US phenotype** | **Gene name** | **Log2FC** | **r** | ***P*-value** |
| --- | --- | --- | --- | --- |
| **Microvascular**  **perfusion** |  |  |  |  |
| **RBF** |  |  |  |  |
| SLCO1B3 | Solute carrier organic anion transporter family member 1B3 | 4.49 | 0.28 | *P <* 0.01 |
| AMACR | Alpha-methylacyl-CoA racemase | 2.77 | 0.27 | *P <* 0.01 |
| SIM2 | SIM bHLH transcription factor 2 | 1.79 | 0.27 | *P <* 0.01 |
| GCNT1 | Glucosaminyl (N-acetyl) transferase 1 | 1.75 | 0.29 | *P <* 0.01 |
| PTN | Pleiotrophin | 1.44 | −0.27 | *P <* 0.01 |
| CTNND2 | Catenin delta 2 | 1.18 | −0.31 | *P <* 0.01 |
| SNAI2 | Snail family transcriptional repressor 2 | −1.86 | −0.32 | *P <* 0.01 |
| SRD5A2 | Steroid 5 alpha-reductase 2 | −1.81 | −0.39 | *P <* 0.01 |
| MAL | Mal, T cell differentiation protein | −1.45 | −0.25 | *P <* 0.01 |
| RND3 | Rho family GTPase 3 | −1.44 | −0.28 | *P <* 0.01 |
| LAMB1 | Laminin subunit beta 1 | −1.41 | −0.26 | *P <* 0.01 |
| CAV1 | Caveolin 1 | −1.30 | −0.28 | *P <* 0.01 |
| MMP16 | Matrix metallopeptidase 16 | −1.16 | −0.28 | *P <* 0.01 |
| SRPX | Sushi repeat containing protein X-linked | −1.23 | −0.39 | *P <* 0.01 |
| ITGB3 | Integrin subunit beta 3 | −1.2 | −0.28 | *P <* 0.01 |
| BMP2 | Bone morphogenetic protein 2 | −1.12 | −0.30 | *P <* 0.01 |
| **PEAK** |  |  |  |  |
| MYC | MYC proto-oncogene, bHLH transcription factor | 1.96 | −0.22 | *P <* 0.01 |
| BIRC5 | Baculoviral IAP repeat containing 5 | 1.60 | −0.27 | *P <* 0.01 |
| CDK5R1 | Cyclin dependent kinase 5 regulatory subunit 1 | 1.50 | −0.24 | *P <* 0.01 |
| POU5F1B | POU class 5 homeobox 1B | 1.10 | −0.26 | *P <* 0.01 |
| **TTP** |  |  |  |  |
| MYC | MYC proto-oncogene, bHLH transcription factor | 1.96 | −0.27 | *P <* 0.01 |
| CDK5R1 | Cyclin dependent kinase 5 regulatory subunit 1 | 1.50 | −0.33 | *P <* 0.01 |
| FASN | Fatty acid synthase | 1.39 | −0.24 | *P <* 0.01 |
| **RBV** |  |  |  |  |
| MAK | Male germ cell associated kinase | 1.40 | 0.27 | *P <* 0.01 |
| RND3 | Rho family GTPase 3 | −1.44 | −0.24 | *P <* 0.01 |

**Table S5.** Gene ontology functional enrichment of the DEGs according to the three key features

| **Functional annotation** | **Genes** | ***P-*adj** |
| --- | --- | --- |
| cd1-GLRLM-GLV |  |  |
| Apoptotic signaling pathway | SNAI2, CAV1, FHIT | 0.01 |
| Negative regulation of epithelial cell proliferation | SNAI2, CAV1 | < 0.01 |
| Androgen biosynthetic process | SRD5A2 | < 0.01 |
| Cell migration | SNAI2, CAV1, RND3 | < 0.01 |
| Gland development | SNAI2, CAV1 | < 0.01 |
| Male genitalia development | SRD5A2 | < 0.01 |
| Androgen metabolic process | SRD5A2 | < 0.01 |
| Negative regulation of lipid biosynthetic process | SNAI2 | 0.01 |
| Lipid biosynthetic process | SNAI2, SRD5A2 | 0.01 |
| Lipid storage | CAV1 | 0.02 |
| Male gonad development | SRD5A2 | 0.04 |
| Regulation of muscle system process | CAV1 | 0.06 |
| cd1-GLSZM-SZLGE |  |  |
| Negative regulation of epithelial cell proliferation | SNAI2, CAV1, PTN | < 0.01 |
| Epithelial cell proliferation | SNAI2, LAMB1, PTN, CAV1 | < 0.01 |
| Regulation of cell proliferation | SNAI2, LAMB1, SRPX, PTN, CAV1, CCND2 | 0.01 |
| Negative regulation of cell proliferation | SNAI2, SRPX, PTN, CAV1 | 0.02 |
| Response to lipid | SNAI2, PTN, CAV1 | 0.01 |
| RBF |  |  |
| Regulation of epithelial cell proliferation | SNAI2, LAMB1, CAV1, BMP2, ITGB3 | < 0.01 |
| Epithelial cell proliferation | SNAI2, LAMB1, CAV1, BMP2, ITGB3 | < 0.01 |
| Regulation of cell migration | SNAI2, LAMB1, CAV1, BMP2, ITGB3 | 0.01 |
| Positive regulation of epithelial cell proliferation | LAMB1, CAV1, ITGB3 | 0.01 |
| Negative regulation of lipid biosynthetic process | SNAI2, BMP2 | 0.02 |
| Lipid storage | CAV1, ITGB3 | < 0.01 |
| Lipid biosynthetic process | SNAI2, BMP2, AMACR, SRD5A2 | < 0.01 |
| Negative regulation of lipid metabolic process | SNAI2, BMP2 | < 0.01 |
| Lipid metabolic process | SNAI2, CAV1, BMP2, AMACR, SRD5A2 | 0.04 |
| Negative regulation of epithelial cell proliferation | SNAI2, CAV1 | 0.03 |
| Androgen biosynthetic process | SNAI2 | 0.05 |
| Male genitalia development | SRD5A2 | 0.02 |
| Androgen metabolic process | SRD5A2 | 0.02 |
| Male gonad development | SRD5A2 | 0.17 |

**Table S6.** KEGG pathway of the differentially expressed genes according to the three key features

| **Functional annotation** | **Genes** | ***P-*value** |
| --- | --- | --- |
| cd1-GLRLM-GLV |  |  |
| Steroid hormone biosynthesis | SRD5A2 | 0.03 |
| Adhesion junction | SNAI2 | 0.03 |
| Bacterial invasion of epithelial cells | CAV1 | 0.04 |
| Prostate cancer | SRD5A2 | 0.05 |
| Hippo signaling pathway | SNAI2 | 0.07 |
| Proteoglycans in cancer | CAV1 | 0.09 |
| cd1-GLSZM-SZLGE |  |  |
| Focal adhesion | CCND2, LAMB1, CAV1 | < 0.01 |
| Hippo signaling pathway | CCND2 | < 0.01 |
| Wnt signaling pathway | W1F1 | < 0.01 |
| MicroRNAs in cancer | CCND2, MMP16 | < 0.01 |
| p53 Signaling pathway | CCND2 | 0.06 |
| Pathways in cancer | CCND2, LAMB1 | 0.07 |
| Prostate cancer | SRD5A2 | 0.08 |
| Transcriptional misregulation in cancer | CCND2 | 0.21 |
| RBF |  |  |
| Focal adhesion | LAMB1, CAV1, ITGB3 | < 0.01 |
| ECM-receptor interaction | LAMB1, ITGB3 | < 0.01 |
| Hippo signaling pathway | BMP2, SNAI2 | 0.01 |
| MicroRNAs in cancer | MMP16, ITGB3 | 0.01 |
| Steroid hormone biosynthesis | SRD5A2 | 0.07 |
| Adhesion junction | SNAI2 | 0.08 |
| PI3K-Akt signaling pathway | LAMB1, ITGB3 | 0.09 |
| TGF-beta signaling pathway | BMP2 | 0.10 |
| Prostate cancer | RD5A2 | 0.11 |

**Table S7.** The AUC performance of the different methods and datasets split into the 10 runs

|  |  | **Random forest** | **Naïve Bayes** | **Support vector machines** |
| --- | --- | --- | --- | --- |
|  |  | **AUC** | **AUC** | **AUC** |
| Clinical data | 1 | 0.77 | 0.85 | 0.92 |
|  | 2 | 0.79 | 0.85 | 0.93 |
|  | 3 | 0.79 | 0.85 | 0.92 |
|  | 4 | 0.79 | 0.84 | 0.92 |
|  | 5 | 0.79 | 0.85 | 0.92 |
|  | 6 | 0.79 | 0.85 | 0.93 |
|  | 7 | 0.78 | 0.85 | 0.92 |
|  | 8 | 0.79 | 0.85 | 0.92 |
|  | 9 | 0.78 | 0.85 | 0.92 |
|  | 10 | 0.79 | 0.85 | 0.93 |
| Average | | 0.785 | 0.846 | 0.922 |
|  |  | **Random forest** | **Naïve Bayes** | **Support vector machines** |
|  |  | **AUC** | **AUC** | **AUC** |
| Transcriptomics | 1 | 0.88 | 0.93 | 0.97 |
|  | 2 | 0.88 | 0.94 | 0.97 |
|  | 3 | 0.86 | 0.94 | 0.97 |
|  | 4 | 0.88 | 0.94 | 0.98 |
|  | 5 | 0.86 | 0.94 | 0.98 |
|  | 6 | 0.89 | 0.93 | 0.97 |
|  | 7 | 0.87 | 0.94 | 0.97 |
|  | 8 | 0.88 | 0.94 | 0.97 |
|  | 9 | 0.88 | 0.94 | 0.97 |
|  | 10 | 0.88 | 0.93 | 0.96 |
| Average | | 0.975 | 0.935 | 0.971 |
|  |  | **Random forest** | **Naïve Bayes** | **Support vector machines** |
|  |  | **AUC** | **AUC** | **AUC** |
| Radiomics | 1 | 0.99 | 0.97 | 0.99 |
|  | 2 | 0.99 | 0.99 | 0.99 |
|  | 3 | 0.99 | 1 | 0.99 |
|  | 4 | 1 | 1 | 0.99 |
|  | 5 | 1 | 0.99 | 0.99 |
|  | 6 | 0.99 | 1 | 0.99 |
|  | 7 | 1 | 0.99 | 0.99 |
|  | 8 | 0.99 | 1 | 0.99 |
|  | 9 | 0.99 | 0.97 | 0.99 |
|  | 10 | 1 | 0.99 | 0.99 |
| Average | | 0.999 | 0.993 | 0.998 |
|  |  | **Random forest** | **Naïve Bayes** | **Support vector machines** |
|  |  | **AUC** | **AUC** | **AUC** |
| Combination | 1 | 1 | 1 | 0.99 |
|  | 2 | 0.99 | 0.99 | 0.99 |
|  | 3 | 1 | 0.99 | 0.99 |
|  | 4 | 1 | 0.99 | 0.99 |
|  | 5 | 1 | 0.99 | 0.99 |
|  | 6 | 0.99 | 0.99 | 0.99 |
|  | 7 | 0.99 | 1 | 0.99 |
|  | 8 | 0.99 | 0.99 | 0.99 |
|  | 9 | 0.99 | 0.95 | 0.99 |
|  | 10 | 1 | 1 | 0.99 |
| Average | | 0.996 | 0.992 | 0.997 |
